# Resistance to the antimitotic drug estramustine is distinct from the multidrug resistant phenotype.

**DOI:** 10.1038/bjc.1991.290

**Published:** 1991-08

**Authors:** L. A. Speicher, V. R. Sheridan, A. K. Godwin, K. D. Tew

**Affiliations:** Department of Pharmacology, Fox Chase Cancer Center, Philadelphia, Pennsylvania 19111.

## Abstract

**Images:**


Br. J. Cancer (1991), 64, 267-273 © Macmillan Press Ltd., 1991

### Resistance to the antimitotic drug estramustine is distinct from the multidrug resistant phenotype

L.A. Speicher[1], V.R. Sheridan[1], A.K. Godwin[2] & K.D. Tew[1]

[1]*Departments of Pharmacology and* [2]*Medical Oncology, Fox Chase Cancer Center, 7701 Burholme Avenue, Philadelphia, Pennsylvania 19111, USA.*

**Summary** Following EMS mutagenesis, three estramustine (EM) resistant DU 145 human prostatic carcinoma cell lines were clonally selected by exposure to incrementally increasing concentrations of the drug. Although only low levels of resistance (approximately 3-fold) were attainable, this resistance was stable in the absence of continuous drug exposure. These EM-resistant clones (EMR 4,9,12) did not exhibit cross resistance to vinblastine, taxol, or adriamycin, and had collateral sensitivity to cytochalasin B. None of the lines had elevated expression of P-glycoprotein mRNA or glutathione S-transferase activity, suggesting a phenotype distinct from the classic multi-drug resistance phenotype. This conclusion was supported further by the observation that two MDR cell lines (FLC mouse erythroleukaemic and SKOV3 human ovarian carcinoma cells) showed sensitivity to EM. Fluorescent activated cell sorting analysis of the effects of EM on cell cycle traverse revealed that at EM concentrations up to 20 $\mu$M an increasing percentage of wild type cells were blocked in $G_2$/M; no such effect occurred in EMR lines. Differential interference contrast microscopy was employed to study EM's effect on mitosis. EMR lines were able to form functional, albeit smaller, spindles at EM concentrations that resulted in chromosomal disorganisation and inhibition of mitotic progression in wild type cells. EMR lines were able to progress through mitosis and cytokinesis at the same rate as untreated cells. Tritiated EM was used to evaluate potential drug uptake/efflux mutations in EMR clones. EMR 4 and 9 incorporate less EM than wild type cells; however, they have significantly decreased cellular volumes. The initial efflux rate constants for EMR clones were greater than for wild type cells. Within 5 min >70% of the drug was lost from resistant cells compared to a 50% loss by the wild type. Although the specific mechanisms of resistance have yet to be defined, the lack of collateral resistance to other MDR/anti-microtubule agents could serve as the basis for the clinical use of EM in combination chemotherapy.

Estramustine (EM) is a chemotherapeutic agent used in the treatment of hormone-refractory, advanced prostate carcinoma (Jonsson *et al.*, 1977; Kuss *et al.*, 1980). As illustrated in Figure 1, EM is an oestradiol linked to nor-nitrogen mustard through a carbamate-ester bond, which is largely responsible for the unusual pharmacologic properties of the drug. The stability of this bond accounts for the long clinical half-life of the parent molecule (Gunnarsson *et al.*, 1981; Gunnarsson *et al.*, 1984). In addition, *in vitro* studies revealed that EM acts independently of its constituent alkylating and estrogen moieties (Tew, 1983; Tew *et al.*, 1983). Specifically, EM binds noncovalently to microtubule associated proteins (MAPs) producing microtubule disassembly, stathmokinesis, and eventual cell death (Hartley-Asp, 1984; Stearns & Tew, 1985; Stearns & Tew, 1988; Kanje *et al.*, 1985). EM also binds a cytoplasmic protein which has been demonstrated in high concentrations in the ventral prostate of both rat and man (Bjork *et al.*, 1982; Forsgren *et al.*, 1979*a*). This protein, subsequently named estramustine binding protein (EMBP) serves to localize EM in prostate tissue and binds the drug with a high affinity and high capacity (Forsgren & Bjork, 1984; Forsgren *et al.*, 1979*b*).

One of the major obstacles to cancer chemotherapy is that tumours develop resistance to drugs used for treatment; therefore, understanding the mechanisms by which this resistance is developed is important for improved clinical applications. Towards this goal, we studied the potential mechanisms by which human prostate carcinoma cell lines (DU 145) become resistant to EM. Estramustine, in common with some MDR drugs, exhibits anti-microtubule effects (Bech-Hansen *et al.*, 1976; Beck, 1983); therefore, we compared EM-resistance properties with the multidrug resistance phenotype, as well as other anti-microtubule agents. Multidrug resistance is characterised by cross-resistance to structurally and functionally unrelated drugs, overexpression of plasma membrane P-glycoprotein, and increased levels of the detoxification enzyme glutathione S-transferase pi (Ling & Thompson, 1974; Ling, 1975; Kartner *et al.*, 1983; Batist *et al.*, 1986). Because microtubules play a major role during mitosis and EM exhibits anti-microtubule properties, we examined the drug's effect on the cell cycle of both sensitive and resistant lines. Other types of drug resistance include altered drug transport mechanisms. Using $^3$H-EM, we investigated the possibility that differential drug uptake and/or efflux characteristics were responsible for the development of EM resistance.

**Figure 1** Chemical structure of estramustine. Estradiol is connected to nor-nitrogen mustard through a carbamate-ester linkage.

The goal of our study was three-fold: (1) to produce EM-resistant DU 145 human prostate carcinoma cells lines, (2) compare EM resistance to that of the MDR phenotype, (3) study the effect of EM on mitosis in both wild type and resistant lines.

### Materials and methods

### *Cell culture*

Human prostatic carcinoma cell lines (DU 145) were cultured in complete media (Dulbecco's Modified Eagle Media supplemented with 4 mM L-glutamine, 1 mM sodium pyruvate, 1% penicillin/streptomycin, and 10% foetal bovine serum (FBS)). Murine erythroleukemic cells sensitive (FLC) and resistant (ARN1, 2 and 3) to adriamycin (cells kindly provided by Haim Tapiero) were grown in RPMI-1640 media supplemented with 10% FBS and 1% penicillin/streptomy-

Correspondence: L.A. Speicher.
Received 10 July 1990; and in revised form 11 March 1991.

cin. Human ovarian carcinoma cells sensitive (SKOV3) and resistant (SKVLB) to vinblastine (cells kindly provided by Victor Ling) were grown in α-MEM supplemented with 15% FBS. SKVLB cells were grown in medium containing 1 μg ml$^{-1}$ vinblastine. All cell lines were incubated at 37°C in a humidified, 5% $CO_2$ atmosphere.

*Establishment of estramustine resistant lines*

DU 145 cells were mutagenised with 300 μg ml$^{-1}$ ethylmethane sulfonate for 36 h, refed with fresh media, and plated onto T-80 dishes in 10 μM EM. Surviving colonies were harvested and passaged at a 1:5 ratio in low (2.5 μM) EM concentrations and maintained in constant exposure to drug. Resistance was achieved through sequential passages in increasing EM concentrations.

*Collateral resistance/sensitivity assays*

Patterns of collateral resistance of wild type and EMR lines to anti-microtubule/MDR agents (adriamycin, cytochalasin B, taxol, vinblastine) were determined using colony forming assays. Cell monolayers were trypsinised from flasks, counted and plated directly into media containing drug at a density of 500 cells/25 cm$^2$ tissue culture flask. Cells were maintained in constant exposure to drug at 37°C in 5% $CO_2$ for 10–12 days. Surviving colonies (>32 cells) were fixed with methanol/acetic acid (90:10), stained with 1% crystal violet, and counted using a Biotran III automatic totalizer (New Brunswick Scientific, Edison, NJ). Percentage of surviving colonies were calculated by comparison to control flasks and plotted as a function of drug concentration. $IC_{50}$ values were calculated from linear regression analysis of plotted values.

*Characterisation assays*

The stability of resistance of the EMR clones to EM was determined by survival assays. Cells were cultured in the absence of drug for 12 weeks (approximately 75 population doublings) and colony forming assays using EM concentrations of 0–20 μM were performed.

To calculate the population doubling times of wild type and EMR clones, cells were plated onto 6 well tissue culture plates at 10% confluency (7–8 × 10$^3$ cells cm$^{-2}$) and cell counts were taken at the same time on five consecutive days. Individual wells were washed 1 × with phosphate buffered saline, trypsinised (0.25% trypsin with .02% EDTA) and counted on a Coulter Counter (Model ZM, Coulter Electronics, Inc., Hialeah, FL). Triplicate wells were counted for each time point and the experiment was repeated three times. Glutathione (GSH) levels and GST activity were determined through methods previously published (Griffith, 1980; Habig et al., 1974).

*Northern blot analysis of RNA from wild type and resistant lines*

Total cellular RNA was isolated by an acid-phenol-chloroform extraction (Chomczynski & Sacchi, 1987), and electrophoresed in 1% agarose - 6% formaldehyde gels. Twenty μg of total RNA was loaded per lane. RNA gels were visualised by ethidium bromide staining to ensure that equivalent amounts of RNA were being analysed. Separated RNA species were transferred to Magna NT membrane filters (Micron Separations, Inc., Westboro, MA) by capillary elution in 10 × SSC and hybridised with $^{32}$P-labelled pCHP1 insert (a 600 bp P-glycoprotein cDNA sequence; Riordan et al., 1985) nick-translated to a specific activity of ~4 × 10$^8$ d.p.m. μg$^{-1}$. Filters were washed twice for 30 min each in 2 × SSC, 0.5% SDS, and 0.1% sodium pyrophosphate at 65°C and twice in 0.2 × SSC, 0.5% SDS, and 0.1% sodium pyrophosphate at 55°C. Autoradiography was performed at −70°C for 1 week.

*FACs*

Fluorescent activated cell sorting was employed to study EM's effect on the cell cycle. Wild type and EMR clones were exposed to EM (0, 2.5, 10, 20 μM) for 36 h and prepared for FACs according to the procedure of Vindelov et al. (1983). Cellular DNA content was analysed on a FACScan (Becton Dickinson Immunocytometry Systems, San Jose, CA). Data are reported as the percentage of a given population in a specific phase of the cell cycle ($G_1$, S, or $G_2$/M).

*Microscopy*

Living cells were prepared for differential interference contrast (DIC) microscopy as follows. Cells were plated onto sterile glass coverslips 3 days prior to use in order to obtain mitotic cells which were relatively flat and thus, optimal for DIC microscopy. The coverslips were placed on slides and buffered with 25 mM HEPES and the slide was maintained at 37°C via an air curtain (Laboratory Products, Inc., Boston, MA). EM-resistant lines were maintained in drug prior to, and throughout the course of observation. Video-enhanced DIC microscopy was performed utilising a DAGE Newvicon (DAGE-MTI, Inc., Michigan City, IN) video camera, Hughes Aircraft model 794 image processor (Hughes Aircraft Co., Los Angeles, CA), Panasonic 794 time/date generator (Panasonic, Inc., Secaucas, NJ) and Sony 5800 3/4 "(Sony Corp., Long Island City, NY) video recorder.

*Analysis of cellular uptake and release of $^3$H-EM*

$^3$H-estramustine (2,4,4,7-$^3$H) was provided by Pharmacia Leo Therapeutics AB (Helsingborg, Sweden). Radiochemical purity was determined by high performance liquid chromatography to be >98%.

Uptake of $^3$H-EM was determined by incubating cells for time periods from 0–24 h at 37°C with 1 μCi ml$^{-1}$ of labelled EM in media containing 2.5 μM cold drug. At each time point, cells were washed with cold PBS, trypsinised and an aliquot removed for cell number determination. Cells were centrifuged through silicon/mineral oil (4:1) at 13,000 *g* for 3 min, media and oils were carefully aspirated and cell pellets were resuspended in a hypertonic lysis buffer. Cells were transferred to scintillation vials with 5.0 ml Ecoscint A scintillation fluid and radioactivity was determined with a Beckman LS 9800 liquid scintillation counter.

Drug efflux was determined following an initial 2 h incubation of cells with $^3$H-EM in the conditions described above. Drug-containing media was aspirated from cells, which were then washed and refed in drug-free media. At time-points from 0 min–4 h post drug-incubation, cells were prepared as described above. All experiments were performed in triplicate and repeated at least twice.

Rate constants for drug efflux were calculated using nonlinear regression analysis.

*Cell volume calculation*

Cell volumes were determined using an electronic particle counter equipped with a size-distribution analyser (Coulter Electronics, Inc., Hialeah, FL). Accumulated data was displayed graphically and digitally and plotted on an XY recorder. Ragweed pollen standard was used to calculate the calibration constant (K). Cell volumes (V) were calculated from the following equation:

$$V(\mu m^3) = (K \times (1/APC) \times (1/AMP) \times T$$

where APC = aperture current, AMP = amplification and T is the modal threshold value determined graphically.

### Results

*Establishment and characterisation of estramustine resistant prostate carcinoma cell lines*

Colony forming assays performed under conditions of continuous exposure to EM determined that wild type cells exhibited an approximate $IC_{90}$ value of 10 μM. EM-resis tant clones (EMR) were established following mutagenesis, through an initial selection of clones surviving 10 μM EM, followed by continued exposure to increasing drug concentrations. Three EMR clones exhibiting an approximate three fold increased resistance to EM compared to the wild type cell line were selected (Figure 2).

The population doubling times for the EMR lines (24–25 ± 2 h) were not significantly different from that of the wild type (26 ± 1 h). In addition, doubling times for EMR clones grown in the presence of 12 μM EM were not significantly different than those for clones grown in the absence of drug (Table I).

The stability of resistance of the EMR clones was determined by colony forming assays after removal of cells from drug for 12 weeks (approximately 75 population doublings). All clones tested maintained a significantly increased level of EM resistance compared to the wild type cells (Table II). In addition, calculated EM $IC_{50}$ and $IC_{90}$ values for EMR clones removed from drug were compared to those of cells maintained in the continual presence of drug. Results of cell growth assays in the presence of variable EM concentrations revealed that the EMR clones removed from drug were able to maintain the same degree of resistance to EM as those continually exposed to drug.

**Figure 2** Estramustine's effect on cell survival of wild type and EMR cell lines. Data are presented as percent cell survival as a function of estramustine concentration. Values represent results of three experiments performed in triplicate ± standard error of the mean. EMR clones exhibit a three fold increased level of resistance compared to wild type. (○) wild type; (△) EMR 4; (□) EMR 9; (■) EMR 12.

**Table I** Calculated doubling times: wild type and EMR cell lines

| *Cell type* | *Doubling time (H)* Drug absence | *Presence of 12 μM EM* |
|---|---|---|
| Wild type | 26.3 ± 1.0 | * |
| EMR 4 | 25.5 ± 2.9 | 25.3 ± 2.4 |
| EMR 9 | 23.5 ± 3.3 | 24.0 ± 0.9 |
| EMR 12 | 21.8 ± 1.8 | 23.7 ± 2.9 |

Cells were plated at a 10% initial seeding density in either 12 μM EM or in the absence of drug. Cell counts were taken at the same time for four consecutive days. Doubling time values calculated represent an $n = 6$ ± standard error of the mean. *Wild type cells were not plated in 12 μM EM, as this would result in 100% cell kill.

**Table II** Stability of estramustine resistant DU 145 cell lines

| *Cell line* | *$IC_{50}$ μM** | *$IC_{90}$ μM* | *Fold resistance* |
|---|---|---|---|
| Wild type | 2.8 (1) | 7.2 (1) | |
| EMR 4 ( + drug) | 14.6 (5.2) | 24.4 (3.4) | 4.3 |
| EMR 4 ( – drug) | 11.7 (4.2) | 21.0 (2.9) | 3.6 |
| EMR 12 ( + drug) | 10.5 (3.8) | 17.9 (2.5) | 3.2 |
| EMR 12 ( + drug) | 9.2 (3.3) | 17.5 (2.4) | 2.9 |

*$IC_{50/90}$ values were calculated by linear regression analysis of results of colony forming assays carried out with continual exposure to drug. Values represent an $n = 3$. Values in parenthesis represent fold resistance compared to drug sensitive parentals. Resistant cell lines were grown in the absence of drug for 12 weeks prior to experiment. Other resistant clones which have not been reported in this manuscript also demonstrate stability to EM.

*Estramustine is distinct from the multi-drug resistance phenotype*

Cross resistance and biological characterisation assays were performed in order to determine if EM resistance was distinct from the MDR phenotype. Survival assays examined the patterns of cross resistance of wild type and EMR cell lines to anti-mitotic/MDR agents. Results from survival assays with vinblastine, adriamycin, taxol, and cytochalasin B are shown in Table III. None of the EMR clones exhibited an increased resistance (compared to wild type) to any of the drugs examined. However, all EMR clones had an approximate three fold increased sensitivity to cytochalasin B. In addition, EMR 12 was approximately twice as sensitive to adriamycin as the wild type line.

Additional support of the hypothesis that EM resistance was distinct from MDR phenotype came from experiments with two carcinoma cell lines known to be part of the MDR phenotype. Friend erythroleukaemic cells (FLC) made resistant to adriamycin (ARN; Tapiero *et al.*, 1984) and human ovarian carcinoma cells (SKOV3) made resistant to vinblastine (SKVLB; Bradley *et al.*, 1989), both demonstrate cross resistance to other anthracylines and vinca alkaloids. The sensitivity of these cells to EM was tested and $IC_{50}$ values for wild type and resistant clones are given in Table IV. None of the resistant clones demonstrated an increased resistance to EM. However, ARN cell lines did exhibit an increased sensitivity to EM.

One of the primary alterations in MDR cells is an increased expression of the MDR1 gene product, plasma membrane P-glycoprotein. Therefore, using Northern blot analysis, EMR clones were tested for increased message levels of P-glycoprotein. As seen in Figure 3, EMR clones did not

**Table III** Cross resistance of estramustine-resistant DU 145 cells toward anti-mitotic/MDR drugs

| | *$IC_{50}$ Values (relative degree of resistance)* Wild type | *EMR 4* | *EMR 9* | *EMR 12* |
|---|---|---|---|---|
| Estramustine (μg/ml) | 2.8 (1) | 9.2 (3.3) | 8.1 (2.9) | 8.8 (3.2) |
| Adriamycin (ng/ml) | 6.9 (1) | 8.7 (1.3) | 6.1 (0.9) | 3.0 (0.4) |
| Cytochalasin B (μg/ml) | 14.0 (1) | 5.0 (0.4) | 5.5 (0.4) | 4.8 (0.3) |
| Taxol (ng/ml) | 2.5 (1) | 1.7 (0.7) | 2.7 (1.1) | 3.1 (1.2) |
| Vinblastine (ng/ml) | 1.8 (1) | 1.6 (0.9) | 1.8 (1.0) | 1.6 (0.9) |

Colony forming assays were performed in continuous exposure to drug as described in Methods. $IC_{50}$ values were calculated by linear regression analysis of results of at least three experiments performed in triplicate. EMR clones do not exhibit increased resistance to any of the drugs tested; however, an increased sensitivity to cytochalasin B is noted.

**Table IV** Estramustine $ID_{50}$ values for MDR cell lines

| *Cell line* | *Resistant to:* | *(fold resistance)* | *EM $ID_{50}$ (μM)* |
|---|---|---|---|
| SKOV3 | Wild Type | | 6.4 |
| [1]SKVLB | Vinblastine | (100 ×) | 6.2 |
| FLC | Wild type | | 4.3 (1) |
| [2]ARN 1 | Adriamycin | (2.5 ×) | 2.7 (0.6) |
| ARN 2 | | (13 ×) | 2.9 (0.7) |
| ARN 3 | | (> 100 ×) | 1.4 (0.3) |

[1,2]SKVLB and ARN cell lines were made resistant to vinblastine and adriamycin, respectively, but demonstrate cross resistance to other anthracyclines and vinca alkaloids (see refs. Tapiero *et al.*, 1984; Bradley *et al.*, 1989). Values in parentheses represent fold increase in resistance compared to wild type cells.

**Figure 3** Northern blot hybridisation with the P-glycoprotein cDNA probe pCHP1. Total RNA was size-fractionated on agarose gels, transferred to nylon membrane filters and hybridised with $^{32}$P-labelled pCHP1 as described in Methods. The ethidium-bromide stained RNA gel (right panel) ensures that equivalent amounts of RNA were analysed. Lanes: (1) human ovarian carcinoma cell line (2780) - negative control; (2) and (3) colon carcinoma cell line HCT15 (sensitive) and HCT15 CP16 (cisplatinum-resistant) both positive controls for MDR 1 RNA expression; (4) wild type; (5), (6), and (7), EMR 4, 9 and 12 respectively. Neither the wild type nor EMR cell lines overexpress MDR-1 RNA.

**Figure 4** Fluorescent activated flow cytometry analysis of estramustine's effect on the cell cycle of wild type **a** and EMR **b**, **c** cell lines: Data are presented as the percentage of cells in a certain cycle phase as a function of estramustine concentration. With increasing EM concentrations, there is a proportional increase in the percent of wild type cells blocked in $G_2$/M. In contrast, the percent of EMR cells in $G_2$/M does not change with increasing EM concentrations. (O) $G_1$; (●) S; (Δ) $G_2$/M.

overexpress P-glycoprotein mRNA. Western blot analysis confirmed these results and showed EMR clones exhibited undetectable levels of P-glycoprotein (data not shown). Because thiols may be an important factor in both MDR and other forms of drug resistance, and EM may be a potential substrate for GSTs (Tew *et al.*, 1986), both GSH levels and total GST activity in drug sensitive and EMR clones were measured. No significant changes in GST or GSH levels were found for EMR cell lines (data not shown).

### *Estramustine's effect on the cell cycle*

Estramustine binds to microtubule associated proteins (MAPs), causing microtubule disassembly and a metaphase block. Using FACs, the effect of EM (36 h exposure) on the cell cycle of wild type and EMR clones was compared. As expected for wild type cells, increased concentrations of EM resulted in an increased build up of cells at the $G_2$/M phase of the cell cycle. Approximately 11% of DU 145 cells were in $G_2$/M when no drug was present. With increasing EM concentrations of 2.5, 10, and 20 μM the percent of cells in $G_2$/M increased to 20%, 38.2%, and 74.3%, respectively. In contrast, the percent of EMR cells in $G_2$/M did not change significantly with exposure to increased EM concentrations. Figure 4 illustrate the percent of wild type and EMR clones in each phase of the cell cycle as a function of EM concentration.

### *Estramustine's effect on the mitotic spindle*

Differential interference contrast (DIC) microscopy was employed to study the effect of EM on mitosis in the EMR cell lines. Drug-sensitive, wild type mitotic cells treated with EM concentrations as low as 2.5 μM lost microtubule organisation resulting in chromosomal disorganisation and inhibition of mitotic progression. Sensitive cells were unable to form functional spindles when treated with EM concentrations of 10–15 μM (data not shown). In contrast, EMR clones treated with 15 μM EM were capable of forming functional, albeit smaller spindles, and progressed through anaphase and cytokinesis at the same rate as untreated cells (Figure 5).

### *Uptake/efflux analysis of $^3$H-EM in EMR and wild type cells*

Tritiated EM was used to investigate potential differential uptake/efflux characteristics of the sensitive and resistant cell lines. Initial uptake of $^3$H-EM by both resistant and sensitive cells was a rapid process. Within 5 min cells contained >50% of the total drug incorporated and by 1 h maximum uptake was reached (Figure 6a). The maximum EM content of the wild type cells was double that of EMR 4 and 9; however, the modal cell volumes of the EMR clones was only 45–50% of the wild type line. Both resistant and sensitive cells lines followed a biphasic drug-efflux profile; however, a marked difference in their rate constants was observed (Figure 6b). EMR 4, 9 and 12 had much greater initial efflux rate constants compared to the wild type. Within 5 min, the EMR clones lost 70–75% of the drug compared to a 50% loss in the wild type line. The secondary phase of efflux of all cell lines was much slower with half lives ranging from 200–330 min.

### Discussion

The unique interactions of EM with MAPs serve as a basis for comparison with other anti-microtubule agents with respect to drug resistance. In the studies carried out to this time, most cell lines resistant to microtubule active drugs fall into two categories: (1) efflux mutants (i.e., cells expressing the multidrug-resistant phenotype) (Ling, 1974; for review see Schibler and Cabral, 1985), (2) mutants exhibiting tubulin subunit alterations (Cabral & Barlow, 1989). The majority of the studies performed have been with Chinese hamster ovary cells, which fall into the MDR category. In order to compare EM with other anti-microtubule/MDR agents, we produced EM-resistant DU 145 clones (EMR). Levels of attainable EM resistance were not greater than three fold.

These low resistance levels were achievable only after multiple rounds of selection in increasing concentrations of drug and required a period of over a year to acquire. Although single-round selections typically yield 2–3 fold drug resistance in microtubule mutants, multiple rounds of selection have resulted in much higher levels of resistance to the selecting drug (Schibler & Cabral, 1985). In addition to the low level of achievable EM resistance, a low mutation frequency ($<10^{-6}$) was observed for the clones. These results, as well as the observation that the EMR lines are stable to resistance when removed from drug, suggest that some type of genetic alteration rather than an induction phenomenon may be contributing to drug resistance.

Because of the important role that MDR plays in determining preclinical response to certain anti-cancer drugs, we examined whether or not the EMR clones demonstrated resistance mechanisms characteristic of this phenotype. Results from a series of collateral resistance and biological characterisation assays indicate that EM resistance is distinct from the MDR phenotype. EMR clones exhibit no cross resistance to other anti-mitotic/MDR agents including adriamycin, cytochalasin B, taxol and vinblastine. In addition, two cell lines known to be part of the MDR phenotype, SKVLB

**Figure 5** Differential interference contrast microscopy pictures of mitotic figures in EMR cells treated with 16 μM EM at anaphase onset. **a** metaphase, **b** anaphase A, **c** anaphase B, **d** telophase. The time required for cells to progress through anaphase, telophase, and cytokinesis was not effected by EM treatment. Bar = 10 μM.

**Figure 6** Time courses of uptake **a** and release **b** of $^3$H-EM by wild type (O) and EMR (Δ, □, ■) cells. EM uptake is expressed as nmoles $10^{-6}$ cells. All cell lines reached maximum drug uptake by one hour. EMR 4 (Δ) and 9 (□) incorporate less total drug and have smaller cellular volumes than wild type. Drug efflux profile for wild type cells **b** is presented as % maximum drug incorporated at time zero. The efflux rate constants, determined by nonlinear regression analysis, are much greater for EMR clones than wild type cells. Each data point represents the mean of two experiments done in triplicate.

and ARN, do not exhibit increased resistance to EM. Finally, EMR clones do not express increased mRNA or protein levels of P-glycoprotein. These results led us to conclude that the EMR clones do not display the MDR phenotype. Overexpression of GST isozymes have been implicated in acquired drug resistance (Wang & Tew, 1985) including the MDR phenotype (Batist *et al.*, 1986; Schisselbauer *et al.*, 1989). Thiol-containing molecules, such as glutathione, have been shown to have a critical role in maintaining microtubule structure and organisation of the mitotic spindle during cell division (Kimura, 1973; Onefelt, 1983; Tew *et al.*, 1985). EM may be a substrate for GST and has been shown previously to influence GSH levels and GST activity in wild type DU 145 cells (Tew *et al.*, 1986). However, we found that the selection pressures producing a stable EM-resistant phenotype do not result in a significantly altered expression of GST or modified intracellular GSH.

Vinblastine and taxol are known to elicit anti-microtubule effects through their direct interaction with tubulin (Bryan, 1971; Schiff *et al.*, 1979). The fact that the EMR clones remain sensitive to both microtubule stabilising as well as destabilising agents suggests that these cells do not express a tubulin alteration (Schiff & Horwitz, 1980). Of interest is the increased sensitivity to cytochalasin B expressed by the EMR lines. In contrast to tubulin targeting agents, cytochalasin B binds to actin and affects microfilament assembly (MacLean-Fletcher & Pollard, 1980). Thus, an increased sensitivity of EMR lines to cytochalasin B suggests a possible alteration in microtubule/microfilament interactions not seen in wild type cells. It has been shown that MAPs are involved in microtubule/microfilament interactions; therefore, an alteration in a MAP may be responsible for the observed increased sensitivity to cytochalasin B of EMR lines (Griffith & Pollard, 1978; Selden & Pollard, 1983).

Frequently, the cytotoxic consequences of anti-microtubule drugs will be manifested through effects on the mitotic spindle, since the microtubule component making up this structure demonstrates increased sensitivity to the drugs. EM has also been demonstrated to affect cells during mitosis, causing a mitotic arrest in both DU 145 and PC-3 prostate cancer lines (Hartley-Asp, 1984). We have recently shown that EM inhibits metaphase to anaphase transition, reduced spindle pole elongation and delays onset of cytokinesis in wild type cells (Sheridan *et al.*, 1991). Utilising FACs in conjunction with light microscopy, we compared the effect of EM on the cell cycle of the wild type and resistant cells. Results from FACs analysis revealed that an increased percentage of wild type cells were blocked in the $G_2$/M phase of the cell cycle in response to increasing EM concentrations. These findings are consistent with the reported stathmokinetic effects of the drug (Tew & Hartley-Asp, 1984). In contrast, EM concentrations up to 20 $\mu$M did not result in an increased percentage of EMR cells blocked in $G_2$/M. Moreover, population doubling times of the EMR lines were not significantly different from wild type; although, the trend was somewhat shorter. In addition, wild type cells had nearly double the DNA content of the EMR clones, suggesting that selection favoured cells with reduced DNA content. Microscopic observations of mitotic EMR cells (Figure 5) revealed that they have a smaller mitotic spindle apparatus. We hypothesise that the reduced chromosome number (unpublished observations) of the EMR clones is related to the smaller mitotic spindle, and perhaps reflects a propensity for cells with altered spindle components to survive the EM challenge. Of interest, MAPs have been shown to stimulate DNA synthesis *in vitro*, suggesting a role for these proteins in the regulation of DNA replication (Shioda *et al.*, 1989). Cellular DNA is not a target for EM (Tew *et al.*, 1983; Hartley-Asp, 1984); however, recent studies showed nuclear uptake of the drug (Hartley-Asp & Kruse, 1986). The authors report that EM binds to the nuclear protein matrix, possibly through interactions with a MAP-like protein. Thus, one may speculate that EM binding to nuclear MAPs indirectly interferes with DNA synthesis, and resistant cells have adapted through a decreased DNA content with concomitantly fewer chromosomes.

Alterations in drug transport mechanisms are correlated with the development of resistance. The possibility that EMR clones were either drug uptake or efflux mutants was examined using $^3$H-EM. Estramustine's ability to freely cross cellular membranes indicates little role for altered mechanisms of uptake, such as energy-dependent or carrier-mediated, in the development of resistance. Our results confirm this. The rapid incorporation of EM into wild type and EMR cells is consistent with reported patterns of EM uptake in human prostate cancer (1013L) and HeLa $S_3$ cell lines (Kruse & Hartley-Asp, 1989). Similar to our findings, the authors demonstrated 50–60% EM uptake within 5 min and maximum uptake by 1 h. The maximum EM content of two of the resistant clones was only half that of the wild type line. However, the modal cellular volumes of these clones was also only 50% of the wild type. Thus, we conclude that altered drug uptake is not responsible for the development of EM resistance. Results from drug efflux studies indicate that the EMR clones do have altered patterns of EM extrusion. All three resistant clones had much greater initial efflux rate constants than wild type cells. The exact mechanisms responsible for the enhanced EM efflux from resistant cells remains to be elucidated; however, we have definitively demonstrated that P-glycoprotein is not expressed in the cells. We cannot conclude at this time that the enhanced EM efflux from the EMR cells has a role in their development of resistance. The wild type cells may have increased levels of EM binding protein or possibly sequester the drug in organelles such as vesicles. Either of these events would result in increased drug accumulation and hence decreased drug efflux. These hypotheses are currently being investigated.

The lack of cross resistance demonstrated by the EMR lines has significant therapeutic implications and can be the basis for use of EM in combination with other chemotherapeutic agents. Biochemical analysis and discovery of the unique mechanism of action of EM serves as the rationale for its use in combination with anti-microtubule agents. Using a tubulin-binding drug in addition to EM would appear to be a rational approach to enhance cytotoxicity through anti-microtubule activity. This concept is supported by *in vitro* studies which demonstrated that EM enhanced the effect of vinblastine on microtubule disassembly in malignant mouse and DU 145 cell lines (Mareel *et al.*, 1988). This pre-clinical rationale, together with the non-overlapping host toxicities of EM and vinblastine create an encouraging basis for evaluation of this combination in human diseases. An early Phase II trial escalating vinblastine to dose-limiting toxicities produced equivocal results (van Belle *et al.*, 1988). We have initiated a Phase II study where EM doses are escalated, while maintaining vinblastine at non-toxic levels. From the pre-clinical data, there is no reason to assume that resistance to one agent will be accompanied by collateral resistance to the other.

The authors would like to thank Donna Bunch for the preparation of this manuscript.

### References

BATIST, G., TULPULE, A., SINHA, B.K., KATKI, A.G., MYERS, C.E. & COWAN, K.H. (1986). Overexpression of a novel anionic glutathione transferase in multidrug-resistant human breast cancer cells. *J. Biol. Chem.*, **261**, 15544.

BECH-HANSEN, N.T., TILL, J.E. & LING, V. (1976). Pleiotropic phenotype of colchicine-resistant CHO cells: cross-resistance and collateral sensitivity. *J. Cel. Physiol.*, **88**, 23.

BECK, W.T. (1983). Vinca alkaloid-resistant phenotype in cultured human leukemic lymphoblasts. *Cancer Treat. Rep.*, **67**, 875.

BJORK, P., FORSGREN, B., GUSTAFSSON, J.-A., PAUSETTE, A. & HOGBERG, B. (1982). Partial characterization and 'quantitation' of a human prostatic estramustine-binding protein. *Cancer Res.*, **42**, 1935.

BRADLEY, G., NAIK, M. & LING, V. (1989). P-glycoprotein expression in multidrug-resistant human ovarian carcinoma cell lines. *Cancer Res.*, **49**, 2790.

BRYAN, J. (1971). Vinblastine and microtubules. I. Induction and isolation of crystals from sea urchin oocytes. *Exp. Cell Res.*, **66**, 129.

CABRAL, F. & BARLOW, S. (1989). Mechanisms by which mammalian cells acquire resistance to drugs that affect microtubule assembly. *FASEB J.*, **3**, 1593.

CHOMCZYNSKI, P. & SACCHI, N. (1987). Single-step method of RNA isolation by acid guanidinium thiocyanate-phenol-chloroform extraction. *Anal. Biochem.*, **162**, 156.

FORSGREN, B., BJORK, P., CARLSTROM, K., GUSTAFSSON, J.-A., PAUSETTE, A. & HOGBERG, B. (1979*a*). Purification and distribution of a major protein in the rat prostate that binds estramustine, a nitrogen mustard derivative of estradiol-17$\beta$. *Proc. Natl Acad. Sci. USA*, **76**, 3149.

FORSGREN, B., GUSTAFSSON, J.-A., PAUSETTE, A. & HOGBERG, B. (1979*b*). Binding characteristics of a major protein in rat ventral prostate cytosol that interacts with estramustine, a nitrogen mustard derivative of 17$\beta$-estradiol. *Cancer Res.*, **39**, 5155.

FORSGREN, B. & BJORK, P. (1984). Specific binding of estramustine to prostatic proteins. *Urology (Suppl.)*, **23**, 34.

GRIFFITH, L.M. & POLLARD, T.D. (1978). Evidence for actin filament microtubule interaction mediated by microtubule-associated proteins. *J. Cell Biol.*, **78**, 958.

GRIFFITH, O.W. (1980). Determination of glutathione and glutathione disulfide using glutathione reductase and 2-vinylpyridine. *Anal. Biochem.*, **106**, 207.

GUNNARSSON, P.O., FORSHELL, G.P., FRITJOFSSON, A. & NORLEN, B.J. (1981) Plasma concentrations of estramustine phosphate and its major metabolites in patients with prostatic carcinoma treated with different doses of estramustine phosphate (Estracyt). *Scand. J. Urol. Nephrol.*, **15**, 201.

GUNNARSSON, P.O., ANDERSSON, S.B., JOHANSSON, S.A., NILSSON, T. & PLYM-FORSHELL, G. (1984). Pharmacokinetics of estramustine phosphate (Estracyt) in prostatic cancer patients. *Eur. J. Clin. Pharmac.*, **26**, 113.

HABIG, W.H., PABST, M.J. & JACKOBY, W.B. (1974). Glutathione S-transferase A: the first enzymatic step in mercapturic acid formation. *J. Biol. Chem.*, **249**, 7130.

HARTLEY-ASP, B. (1984). Estramustine-induced mitotic arrest in two human prostatic carcinoma cell lines, DU 145 and PC-3. *Prostate*, **5**, 93.

HARTLEY-ASP., B. & KRUSE, K. (1986). Nuclear protein matrix as a target for estramustine-induced cell death. *The Prostate*, **9**, 387.

JONSSON, G., HOGBERG, B. & NILSSON, T. (1977). Treatments of advanced prostatic carcinoma with estramustine phosphate (Estracyt). *Scand. J. Urol. Nephrol.*, **11**, 231.

KANJE, M., DEINUM, J., WALLIN, M., EKSTROM, P., EDSTROM, A. & HARTLEY-ASP, B. (1985). Effect of estramustine phosphate on the assembly of isolated bovine brain microtubules and fast axonal transport in the frog sciatic nerve. *Cancer Res.*, **45**, 2234.

KARTNER, N., SHALES, M., RIORDAN, J.R. & LING, V. (1983). Daunorubicin resistant Chinese Hamster Ovary cells expressing multidrug resistance and a cell-surface P-glycoprotein. *Cancer Res.*, **43**, 4413.

KIMURA, I. (1973). Further evidence of the similarity of microtubule protein from mitotic apparatus and sperm tail of the sea urchin, as a substrate in thiol:disulfide exchange reaction. *Expl. Cell Res.*, **79**, 445.

KRUSE, E. & HARTLEY-ASP, B. (1989). A time study on the uptake of estramustine into prostatic tumor 1013 L cells *in vitro*. *Biochem. Pharm.*, **38**, 702.

KUSS, R., KHORY, S., RICHARD, F., FOURCADE, F., FRANTZ, P. & CAPELLE, J.P. (1980). Estramustine phosphate in the treatment of advanced prostatic cancer. *Br. J. Urol.*, **52**, 29.

LING, V. & THOMPSON, L.H. (1974). Reduced permeability in CHO cells as a mechanism of resistance to colchicine. *J. Cell Physiol.*, **83**, 103.

LING, V. (1975). Drug resistance and membrane alteration in mutants of mammalian cells. *Can. J. Genet. Cytol.*, **17**, 503.

MACLEAN-FLETCHER, S. & POLLARD, T.D. (1980). Mechanism of action of cytochalasin B on actin. *Cell*, **20**, 329.

MAREEL, M.M., STORME, G.A., DRAGONETT, C.H. & 5 others (1988). Anti-invasive activity of estramustine on malignant $MO_4$ cells and on DU 145 human prostate carcinoma cells *in vitro*. *Cancer Res.*, **48**, 1842.

ONEFELT, A. (1983). Spindle disturbances in mammalian cells. I. Changes in the quantity of free sulfhydryl groups in relation to survival and C-mitosis in V-79 Chinese hamster cells after treatment with colcemid, idamide, carbaryl and methylmercury. *Chem. Biol. Interact.*, **46**, 201.

RIORDAN, J.R., DEUCHARS, K., KARTNER, N., ALON, N, TRENT, J. & LING, Y. (1985). Amplification of P-glycoprotein genes in multidrug-resistant mammalian cell lines. *Nature*, **316**, 817.

SCHIBLER, M.J. & CABRAL, F. (1985). Microtubule mutants. In *Molecular Cell Genetics*, Gottesman, M. (ed.) p. 669. John Wiley & Sons: New York.

SCHIFF, P.B., FANT, J. & HORWITZ, S.B. (1979). Promotion of microtubule assembly *in vitro* by taxol. *Nature*, **277**, 665.

SCHIFF, P.B. & HORWITZ, S.B. (1980). Taxol stabilizes microtubules in mouse fibroblast cells. *Proc. Natl Acad. Sci. USA*, **77**, 1561.

SCHISSELBAUER, J., CRESCIAMANNO, M., D'ALESSANDRO, N., CLAPPER, M.L., TAPIERO, H. & TEW, K.D. (1989). Glutathione, glutathione S-transferases and related redox enzymes in adriamycin resistant cell lines with a multidrug resistant phenotype. *Cancer Commun.*, **1**, 133.

SELDEN, S.C. & POLLARD, T.D. (1983). Phosphorylation of microtubule-associated proteins regulates their interaction with actin filaments. *J. Biol. Chem.*, **258**, 7064.

SHERIDAN, V.R., SPEICHER, L.A. & TEW, K.D. (1991). The effects of estramustine on mitotic progression in DU 145 human prostatic carcinoma cells. *Eur. J. Cell Biol.*, **54**, 268.

SHIODA, M., MUROFUSHI, H., MURAKAMI-MUROFUSHI, K. & SAKAI, H. (1989). Microtubule-associated protein-2 stimulates DNA synthesis catalyzed by the nuclear matrix. *Biochem. & Biophys. Res. Commun.*, **159**, 834.

STEARNS, M.E. & TEW, K.D. (1985). Anti-microtubule effects of estramustine, an antiprostatic tumor drug. *Cancer Res.*, **45**, 3891.

STEARNS, M.E. & TEW, K.D. (1988). Estramustine binds MAP-2 to inhibit microtubule assembly *in vitro*. *J. Cell Sci.*, **89**, 331.

TAPIERO, H., MUNCH, J.N., FOURCADE, A. & LAMPIDIS, T.J. (1984). Cross-resistance to rhodamine 123 in adriamycin- and daunorubicin-resistant Friend leukemia cell variants. *Cancer Res.*, **44**, 5544.

TEW, K.D. (1983). The mechanism of action of estramustine. *Semin. Oncol.*, **10**, 21.

TEW, K.D., ERICKSON, L.C., WHITE, G., WANG, A.L., SCHEIN, P.S. & HARTLEY-ASP, B. (1983). Cytotoxicity of estramustine, a steroid-nitrogen mustard derivative, through non-DNA targets. *Molec. Pharmacol.*, **24**, 324.

TEW, K.D. & HARTLEY-ASP, B. (1984). Cytotoxic properties of estramustine unrelated to alkylating and steroid constituents. *Urology*, **23**, 28.

TEW, K.D., KYLE, G., JOHNSON, A. & WANG, A.L. (1985). Carbamoylation of glutathione reductase and changes in cellular and chromosome morphology in a rat cell line resistant to nitrogen mustards but collaterally sensitive to nitrosoureas. *Cancer Res.*, **45**, 2326.

TEW, K.D., WOODWORTH, A. & STEARNS, M.E. (1986). Anti-mitotic properties of estramustine are accompanied by a depletion in intracellular glutathione and an inhibition in glutathione S-transferase. *Cancer Treat. Rep.*, **70**, 715.

WANG, A.L. & TEW, K.D. (1985). Increased glutathione S-transferase activity in a cell line with acquired resistance to nitrogen mustards. *Cancer Treat. Rep.*, **69**, 677.

VAN BELLE, S.I.P., SCHALLER, D., DEWASCH, & STORREL, G. (1988). Broad phase II study of the combination of two microtubule inhibitors: estramustine and vinblastine. *Proc. Amer. Soc. Clin. Oncol.*, **7**, 207.

VINDELOV, L.L., CHRISTENSEN, I.J. & NISSEN, N.I. (1983). A detergent-trypsin method for the preparation of nuclei for flow cytometric DNA analysis. *Cytometry*, **3**, 323.


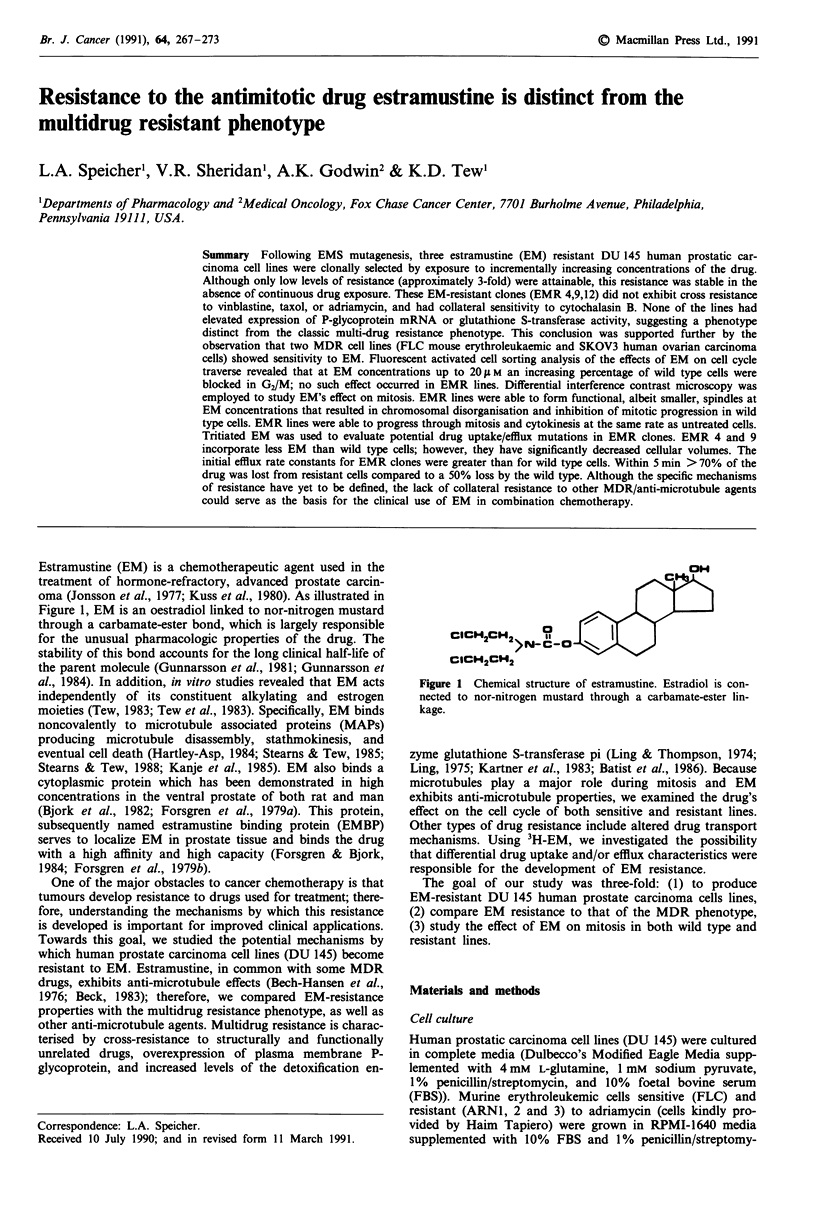

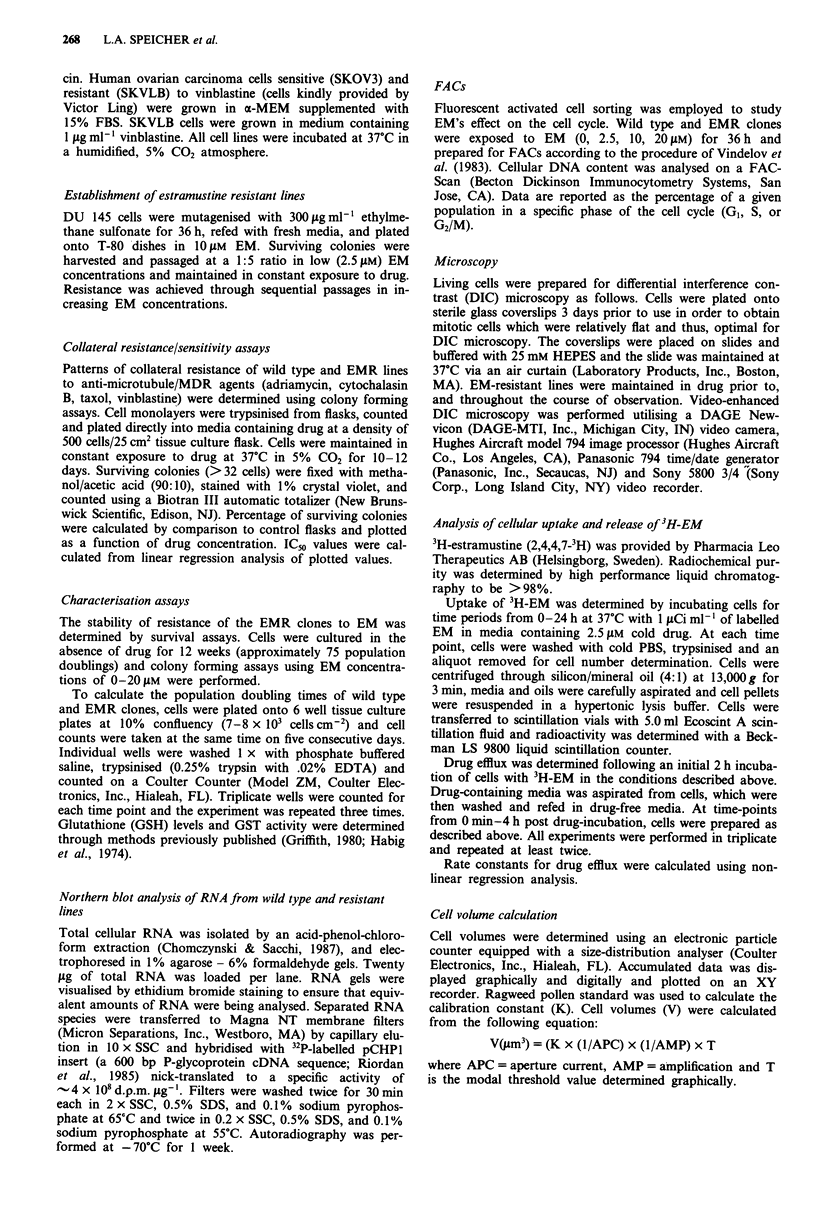

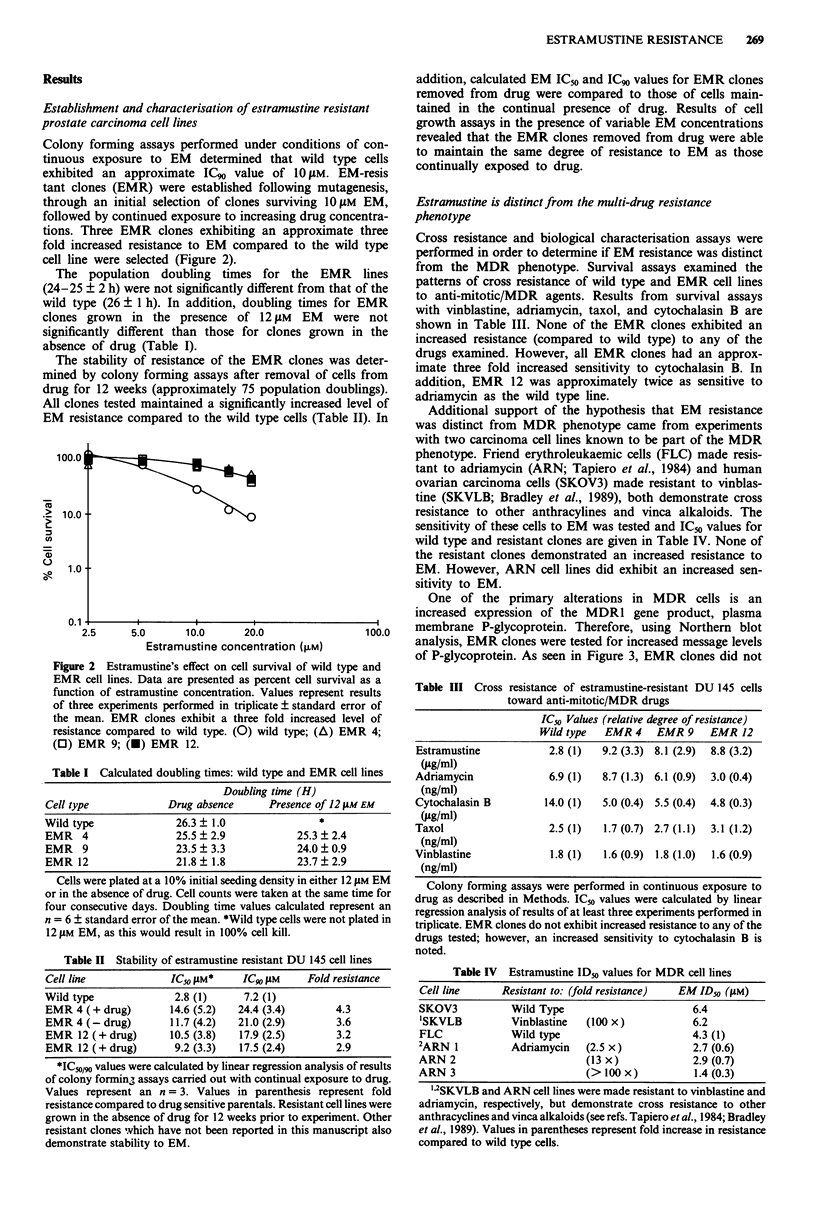

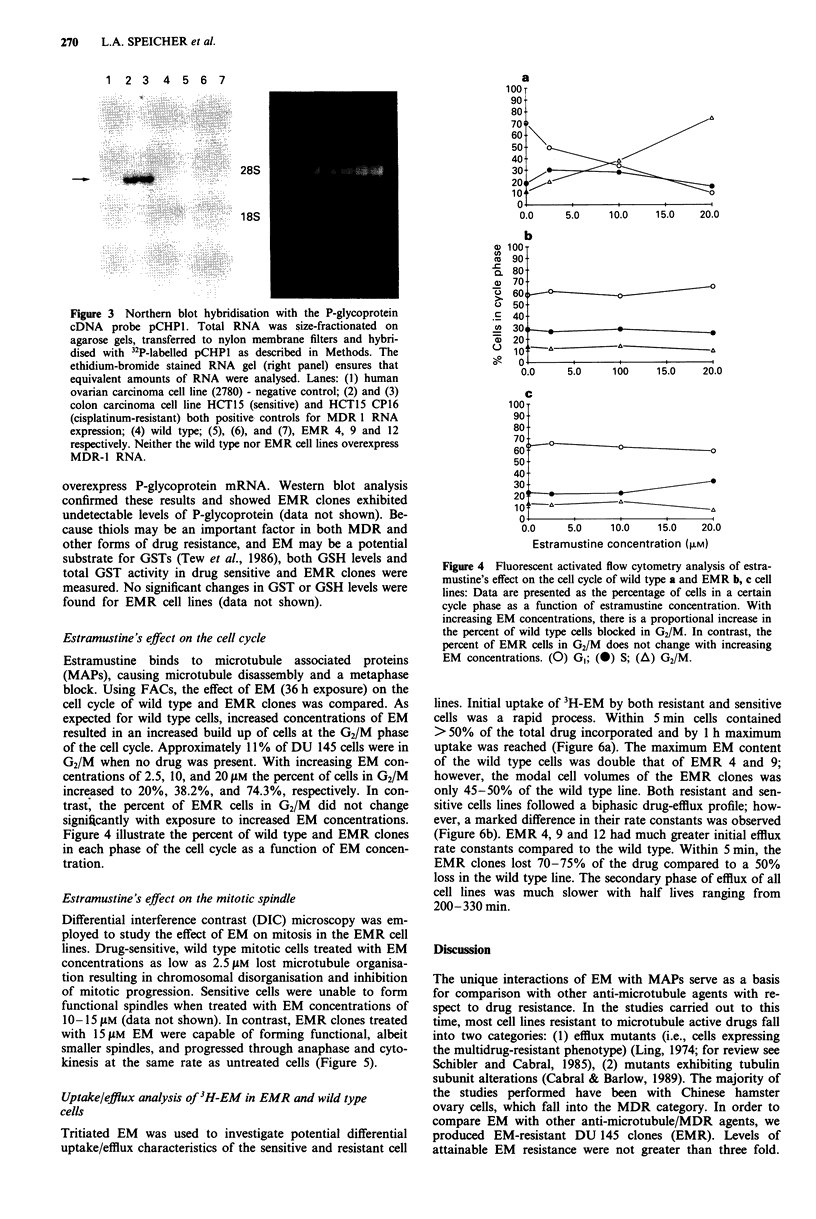

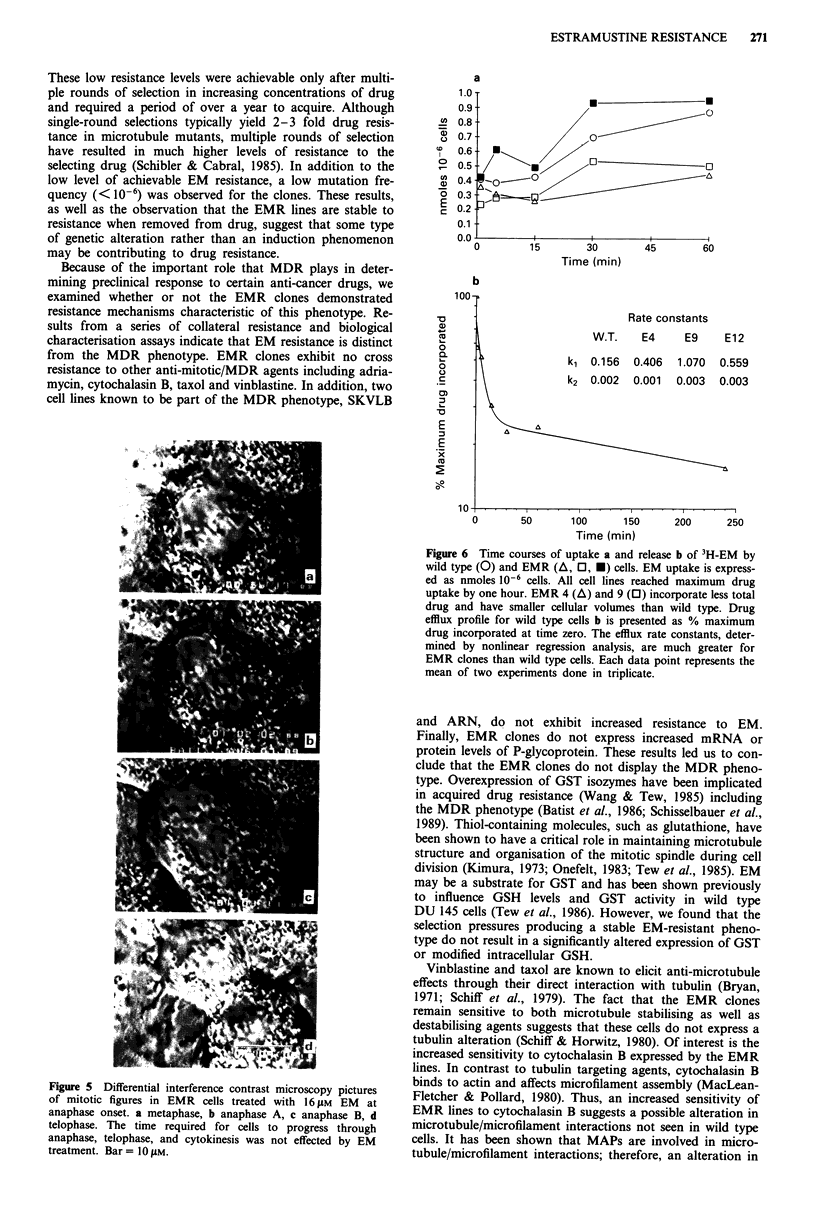

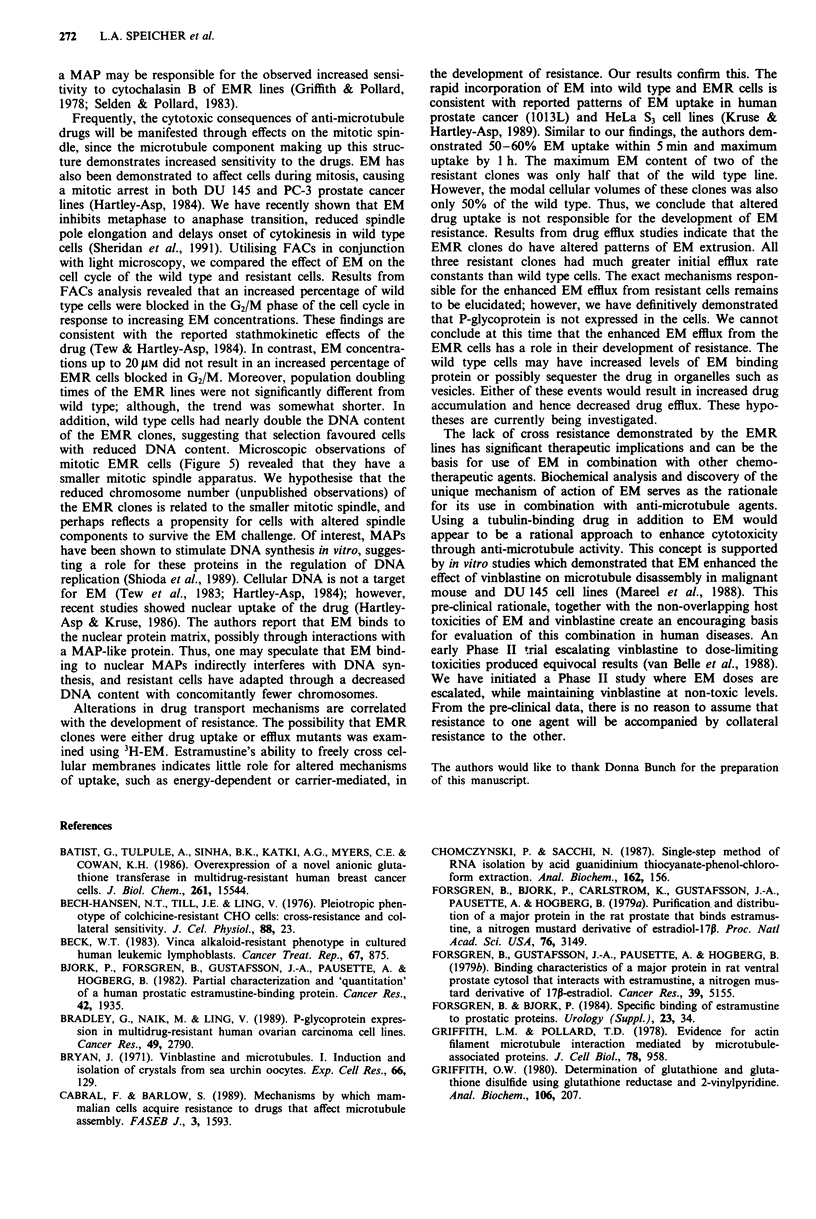

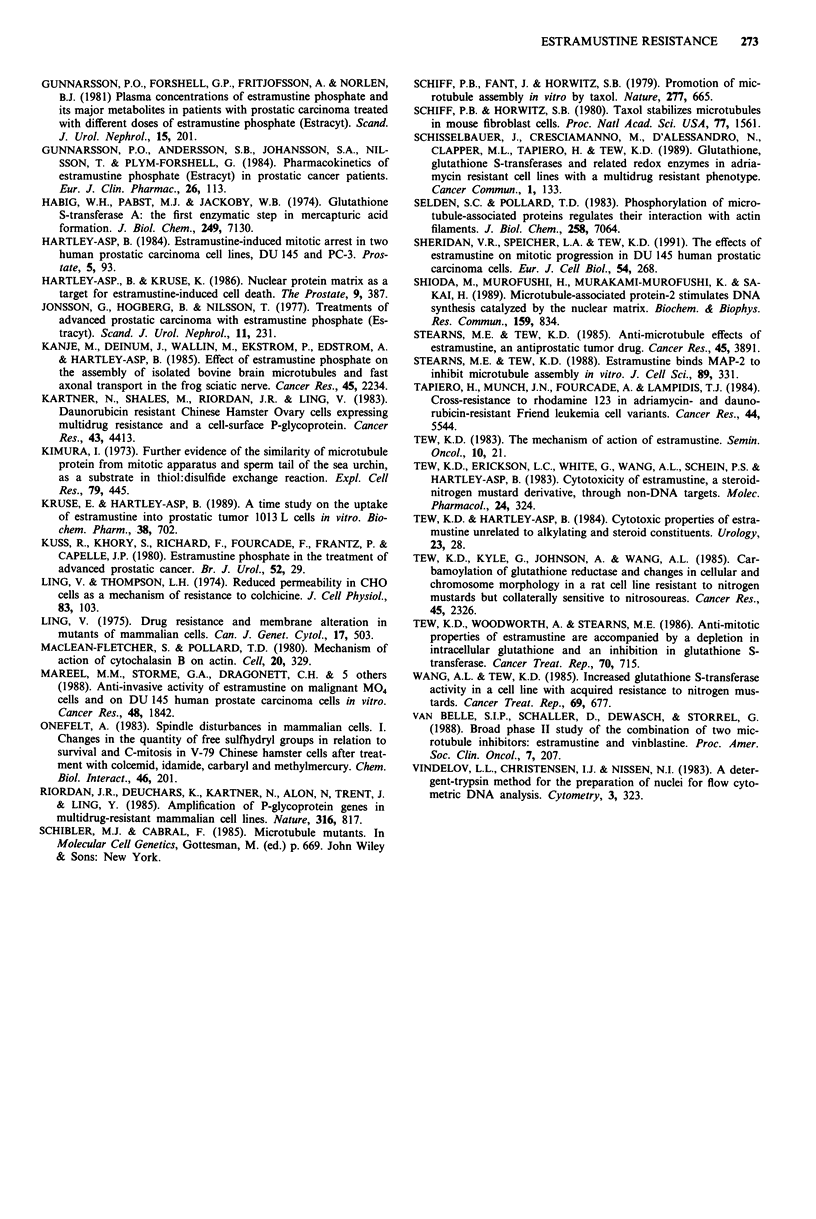


## References

[OCR_00941] Batist G., Tulpule A., Sinha B. K., Katki A. G., Myers C. E., Cowan K. H. (1986). Overexpression of a novel anionic glutathione transferase in multidrug-resistant human breast cancer cells.. J Biol Chem.

[OCR_00947] Bech-Hansen N. T., Till J. E., Ling V. (1976). Pleiotropic phenotype of colchicine-resistant CHO cells: cross-resistance and collateral sensitivity.. J Cell Physiol.

[OCR_00952] Beck W. T. (1983). Vinca alkaloid-resistant phenotype in cultured human leukemic lymphoblasts.. Cancer Treat Rep.

[OCR_00956] Björk P., Forsgren B., Gustafsson J. A., Pousette A., Högberg B. (1982). Partial characterization and "quantitation" of a human prostatic estramustine-binding protein.. Cancer Res.

[OCR_00962] Bradley G., Naik M., Ling V. (1989). P-glycoprotein expression in multidrug-resistant human ovarian carcinoma cell lines.. Cancer Res.

[OCR_00967] Bryan J. (1971). Vinblastine and microtubules. I. Induction and isolation of crystals from sea urchin oocytes.. Exp Cell Res.

[OCR_00972] Cabral F., Barlow S. B. (1989). Mechanisms by which mammalian cells acquire resistance to drugs that affect microtubule assembly.. FASEB J.

[OCR_00977] Chomczynski P., Sacchi N. (1987). Single-step method of RNA isolation by acid guanidinium thiocyanate-phenol-chloroform extraction.. Anal Biochem.

[OCR_00982] Forsgren B., Björk P., Carlström K., Gustafsson J. A., Pousette A., Högberg B. (1979). Purification and distribution of a major protein in rat prostate that binds estramustine, a nitrogen mustard derivative of estradiol-17 beta.. Proc Natl Acad Sci U S A.

[OCR_00995] Forsgren B., Björk P. (1984). Specific binding of estramustine to prostatic proteins.. Urology.

[OCR_00989] Forsgren B., Gustafsson J. A., Pousette A., Högberg B. (1979). Binding characteristics of a major protein in rat ventral prostate cytosol that interacts with estramustine, a nitrogen mustard derivative of 17 beta-estradiol.. Cancer Res.

[OCR_00999] Griffith L. M., Pollard T. D. (1978). Evidence for actin filament-microtubule interaction mediated by microtubule-associated proteins.. J Cell Biol.

[OCR_01004] Griffith O. W. (1980). Determination of glutathione and glutathione disulfide using glutathione reductase and 2-vinylpyridine.. Anal Biochem.

[OCR_01020] Gunnarsson P. O., Andersson S. B., Johansson S. A., Nilsson T., Plym-Forshell G. (1984). Pharmacokinetics of estramustine phosphate (Estracyt) in prostatic cancer patients.. Eur J Clin Pharmacol.

[OCR_01011] Gunnarsson P. O., Forshell G. P., Fritjofsson A., Norlén B. J. (1981). Plasma concentrations of estramustine phosphate and its major metabolites in patients with prostatic carcinoma treated with different doses of estramustine phosphate (Estracyt).. Scand J Urol Nephrol.

[OCR_01024] Habig W. H., Pabst M. J., Jakoby W. B. (1974). Glutathione S-transferases. The first enzymatic step in mercapturic acid formation.. J Biol Chem.

[OCR_01029] Hartley-Asp B. (1984). Estramustine-induced mitotic arrest in two human prostatic carcinoma cell lines DU 145 and PC-3.. Prostate.

[OCR_01034] Hartley-Asp B., Kruse E. (1986). Nuclear protein matrix as a target for estramustine-induced cell death.. Prostate.

[OCR_01037] Jönsson G., Högberg B., Nilsson T. (1977). Treatment of advanced prostatic carcinoma with estramustine phosphate (Estracyt).. Scand J Urol Nephrol.

[OCR_01042] Kanje M., Deinum J., Wallin M., Ekström P., Edström A., Hartley-Asp B. (1985). Effect of estramustine phosphate on the assembly of isolated bovine brain microtubules and fast axonal transport in the frog sciatic nerve.. Cancer Res.

[OCR_01047] Kartner N., Shales M., Riordan J. R., Ling V. (1983). Daunorubicin-resistant Chinese hamster ovary cells expressing multidrug resistance and a cell-surface P-glycoprotein.. Cancer Res.

[OCR_01053] Kimura I. (1973). Further evidence of the similarity of microtubule protein from mitotic apparatus and sperm tail of the sea urchin, as a substrate in thiol-disulfide exchange reaction.. Exp Cell Res.

[OCR_01059] Kruse E., Hartley-Asp B. (1989). A time study on the uptake of estramustine into prostatic tumour 1013L cells in vitro.. Biochem Pharmacol.

[OCR_01064] Küss R., Khoury S., Richard F., Fourcade F., Frantz P., Capelle J. P. (1980). Estramustine phosphate in the treatment of advanced prostatic cancer.. Br J Urol.

[OCR_01074] Ling V. (1975). Drug resistance and membrane alteration in mutants of mammalian cells.. Can J Genet Cytol.

[OCR_01069] Ling V., Thompson L. H. (1974). Reduced permeability in CHO cells as a mechanism of resistance to colchicine.. J Cell Physiol.

[OCR_01078] MacLean-Fletcher S., Pollard T. D. (1980). Mechanism of action of cytochalasin B on actin.. Cell.

[OCR_01082] Mareel M. M., Storme G. A., Dragonetti C. H., De Bruyne G. K., Hartley-Asp B., Segers J. L., Rabaey M. L. (1988). Antiinvasive activity of estramustine on malignant MO4 mouse cells and on DU-145 human prostate carcinoma cells in vitro.. Cancer Res.

[OCR_01088] Onfelt A. (1983). Spindle disturbances in mammalian cells. I. Changes in the quantity of free sulfhydryl groups in relation to survival and C-mitosis in V79 Chinese hamster cells after treatment with colcemid, diamide, carbaryl and methyl mercury.. Chem Biol Interact.

[OCR_01095] Riordan J. R., Deuchars K., Kartner N., Alon N., Trent J., Ling V. Amplification of P-glycoprotein genes in multidrug-resistant mammalian cell lines.. Nature.

[OCR_01105] Schiff P. B., Fant J., Horwitz S. B. (1979). Promotion of microtubule assembly in vitro by taxol.. Nature.

[OCR_01109] Schiff P. B., Horwitz S. B. (1980). Taxol stabilizes microtubules in mouse fibroblast cells.. Proc Natl Acad Sci U S A.

[OCR_01112] Schisselbauer J. C., Crescimanno M., D'Alessandro N., Clapper M., Toulmond S., Tapiero H., Tew K. D. (1989). Glutathione, glutathione S-transferases, and related redox enzymes in Adriamycin-resistant cell lines with a multidrug resistant phenotype.. Cancer Commun.

[OCR_01119] Selden S. C., Pollard T. D. (1983). Phosphorylation of microtubule-associated proteins regulates their interaction with actin filaments.. J Biol Chem.

[OCR_01124] Sheridan V. R., Speicher L. A., Tew K. D. (1991). The effects of estramustine on metaphase and anaphase in DU 145 prostatic carcinoma cells.. Eur J Cell Biol.

[OCR_01131] Shioda M., Murofushi H., Murakami-Murofushi K., Sakai H. (1989). Microtubule-associated protein-2 stimulates DNA synthesis catalyzed by the nuclear matrix.. Biochem Biophys Res Commun.

[OCR_01135] Stearns M. E., Tew K. D. (1985). Antimicrotubule effects of estramustine, an antiprostatic tumor drug.. Cancer Res.

[OCR_01138] Stearns M. E., Tew K. D. (1988). Estramustine binds MAP-2 to inhibit microtubule assembly in vitro.. J Cell Sci.

[OCR_01142] Tapiero H., Munck J. N., Fourcade A., Lampidis T. J. (1984). Cross-resistance to rhodamine 123 in Adriamycin- and daunorubicin-resistant Friend leukemia cell variants.. Cancer Res.

[OCR_01152] Tew K. D., Erickson L. C., White G., Wang A. L., Schein P. S., Hartley-Asp B. (1983). Cytotoxicity of estramustine, a steroid-nitrogen mustard derivative, through non-DNA targets.. Mol Pharmacol.

[OCR_01158] Tew K. D., Hartley-Asp B. (1984). Cytotoxic properties of estramustine unrelated to alkylating and steroid constituents.. Urology.

[OCR_01163] Tew K. D., Kyle G., Johnson A., Wang A. L. (1985). Carbamoylation of glutathione reductase and changes in cellular and chromosome morphology in a rat cell line resistant to nitrogen mustards but collaterally sensitive to nitrosoureas.. Cancer Res.

[OCR_01148] Tew K. D. (1983). The mechanism of action of estramustine.. Semin Oncol.

[OCR_01170] Tew K. D., Woodworth A., Stearns M. E. (1986). Relationship of glutathione depletion and inhibition of glutathione-S-transferase activity to the antimitotic properties of estramustine.. Cancer Treat Rep.

[OCR_01187] Vindeløv L. L., Christensen I. J., Nissen N. I. (1983). A detergent-trypsin method for the preparation of nuclei for flow cytometric DNA analysis.. Cytometry.

[OCR_01176] Wang A. L., Tew K. D. (1985). Increased glutathione-S-transferase activity in a cell line with acquired resistance to nitrogen mustards.. Cancer Treat Rep.

